# Supplementary material for: General strategy for developing thick-film micro-thermoelectric coolers from material fabrication to device integration
Source: Nat Commun. 2024 May 8;15:3870. doi: 10.1038/s41467-024-48346-6 (PMC11079074; doi:10.1038/s41467-024-48346-6)
Supplement: Supplementary file 1 — Supplementary Information [file 41467_2024_48346_MOESM1_ESM.pdf]

Supplementary Information

General strategy for developing thick-film micro-thermoelectric coolers from material fabrication to device integration

Xiaowen Sun[1,2,3], Yuedong Yan[2,3]*, Man Kang[1,2], Weiyun Zhao[2], Kaifen Yan[2], He Wang[2], Ranran Li[2], Shijie Zhao[2], Xiaoshe Hua[2], Boyi Wang[2], Weifeng Zhang[2]* & Yuan Deng[1,2]*

[1]Research Institute for Frontier Science, Beihang University, Beijing, China.

[2]Key Laboratory of Intelligent Sensing Materials and Chip Integration Technology of Zhejiang Province, Hangzhou Innovation Institute of Beihang University, Hangzhou, China.

[3]These authors contributed equally: Xiaowen Sun, Yuedong Yan.

*e-mail: yuedongyan@buaa.edu.cn; zhangweifeng@buaa.edu.cn; dengyuan@buaa.edu.cn

Supplementary Note 1. Cox-Strack method

The Cox-Strack method was used to measure the out-of-plane electrical conductivity (*σ*) of Bi_2Te_3-based TE thick films and the electrical contact resistivity (r_{ec}) between the thick films and electrodes[1]. Supplementary Figure 5a shows the test structure with the TE thick film sandwiched by a bottom electrode and some circular top contact metal pads with diameters (*d*) of 0.2-2 mm. To be consistent with the contact conditions in the as-integrated thick-film μ-TECs, the surface treatment was performed for the bottom electrode, top contact metal pads, and TE thick films. The 5μmNi/200nmAu bilayer was electroplated on the surface of the thick Cu films as the bottom electrode and top contact pads, and the 1μmNi/200nmAu bilayer was deposited on the entire bottom surface and partial top surface (circular areas with the same size as the top contact pads, steel stencil as the mask) of the TE thick film by magnetron sputtering after an in-situ plasma cleaning. Subsequently, the bottom surface of TE thick film was soldered to the bottom electrode, and the top contact pads were soldered to the corresponding circular Ni/Au bilayer of the thick-film top surface. Finally, a Keithley 2400 digital multimeter was applied to measure the resistance (*R*) between the bottom electrode and top contact pads at 25 °C. The resistance can be expressed by the Cox-Strack equation:

$$R = \frac{1}{d\pi\sigma} arctan \frac{4}{d/t} + \frac{4r_{ec}}{\pi d^2} + R_0 \quad (1)$$

where *t* is the thickness of the TE thick film, and R_0 is the residual resistance other than the TE-film resistance (first term on the right) and top contact resistance (second term on the right). By fitting the *d*-dependence of measured *R* with the Cox-Strack equation, the *σ* and r_{ec} can be extracted (Supplementary Fig. 5b). For the as-prepared N-type Bi_2Te_3 thick film with a thickness of 100 μm, the *σ* and r_{ec} were determined to be 103164.3 $S{\cdot}m^{-1}$ and 1.047×10^{-10} $\Omega{\cdot}m^2$, respectively. For the as-prepared P-type $Sb_{1.5}Bi_{0.5}Te_3$ thick film with a thickness of 100 μm, the *σ* and r_{ec} were determined to be 71315.5 $S{\cdot}m^{-1}$ and 1.051×10^{-10} $\Omega{\cdot}m^2$, respectively.

Furthermore, the d^{-2}-dependence of R is plotted in Supplementary Fig. 5c, where the σ is kept constant and the influence of r_{ec} can be clearly observed. When the r_{ec} is varied to deviate from 1.05×10^{-10} Ω·m^2, the resistance deviates significantly from the measured values. At last, we can put the r_{ec} in a small range from 5.0×10^{-11} to 5.0×10^{-10} Ω·m^2, demonstrating the reliability of the fitting results shown in Supplementary Fig. 5b.

Supplementary Note 2. Optimization of thermoelectric leg height and device fill factor

In order to achieve excellent comprehensive cooling performance for the micro-thermoelectric coolers (μ-TECs), a simplified one-dimensional model was performed to optimize the thermoelectric (TE) leg height and device filling factor. Supplementary Figure 6a presents a TEC device containing a pair of TE legs. The Bi_2Te_3 and $Bi_{0.5}Sb_{1.5}Te_3$ prepared by the powder direct molding method were used as the N-type and P-type TE materials for TEC, respectively. We adopted the out-of-plane TE properties for simulation calculations, assuming that the material properties are temperature-independent. The Seebeck coefficient, thermal conductivity, and electrical conductivity were measured using the portable Seebeck tester, laser flash method, and Cox-Strack method (Supplementary Fig. 5 and Note 1), respectively. All the material parameters are shown in Supplementary Table 2. It is assumed that the TEC operates in an adiabatic environment of 25 °C with an ideal heat dissipation of hot side. Based on the simplified one-dimensional model, the TEC can be equivalent to simple thermal and electrical circuits, as shown in Supplementary Fig. 6b,c. Then, the heat flows induced by the Peltier effect and temperature differences inside the TEC can be expressed as:

$$Q_{cs} = \frac{1}{R_{ts}}(T_c - T_{cs}) \tag{2}$$

$$Q_{cc} = \frac{1}{R_{tc}}(T_{cs} - T_{cl}) \tag{3}$$

$$Q_{cl} = S \cdot T_{cl} \cdot I - \frac{1}{2}I^2R - \frac{1}{R_{tl}}(T_{hl} - T_{cl}) \tag{4}$$

$$Q_{hl} = S \cdot T_{hl} \cdot I + \frac{1}{2}I^2R - \frac{1}{R_{tl}}(T_{hl} - T_{cl}) \tag{5}$$

$$Q_{hc} = \frac{1}{R_{tc}}(T_{hl} - T_{hs}) \tag{6}$$

$$Q_{hs} = \frac{1}{R_{ts}}(T_{hs} - T_h) \tag{7}$$

Here, R_{ts}, R_{tc}, and R_{tl} are the thermal resistance of substrates, thermal contact resistance between the electrodes and TE legs, and thermal resistance of TE legs, respectively. The thermal resistance of electrodes and thermal contact resistance between the substrates and electrodes are neglected. Q_{cs} and Q_{hs} are the heat flows through R_{ts}, Q_{cc} and Q_{hc} are the heat flows through R_{tc}, Q_{cl} is the heat flow out of the cold side of TE legs, and Q_{hl} is the heat flow into the hot side of TE legs. T_c and T_h are the cold-side and hot-side temperatures of device, T_{cs} and T_{hs} are the ceramic-side temperatures of interface contact sections, T_{cl} and T_{hl} the cold-side and hot-side temperatures of TE legs. I, S, and R are the input current, total Seebeck coefficient of TE legs, and total electrical resistance of device, respectively. Some parameters can be further expressed as:

$$R_{ts} = \frac{H_s}{\kappa_s A_s} = \frac{H_s f}{\kappa_s (A_n + A_p)} \tag{8}$$

$$R_{tc} = \frac{r_{tc}}{A_n + A_p} \tag{9}$$

$$R_{tl} = \frac{H}{\kappa_n A_n} + \frac{H}{\kappa_p A_p} \tag{10}$$

$$S = S_p - S_n \tag{11}$$

$$R = R_{en} + R_{ep} + R_{ee} + 4R_{ec} = \frac{H}{\sigma_n A_n} + \frac{H}{\sigma_p A_p} + R_{ee} + 4\frac{r_{ec}}{A_n} \tag{12}$$

Here, S_n, σ_n, κ_n, A_n, and R_{en} (S_p, σ_p, κ_p, A_p, and R_{ep}) are the Seebeck coefficient, electrical conductivity, thermal conductivity, cross-sectional area, and resistance of the N-type (P-type) TE leg, respectively. A_n is assumed to be equal to A_p. R_{ee} and R_{ec} are the electrode resistance and the electrical contact resistance between the electrodes and TE legs, respectively. H_s, κ_s, and A_s are the thickness, thermal conductivity, and surface area of the substrates, respectively. r_{tc} and r_{ec} are the thermal and electrical contact resistivity between the electrodes and TE legs, respectively. H and f are the TE leg height and device filling factor, respectively. When the TEC operates under the steady-state thermal equilibrium condition, there are $Q_c = Q_{cs} = Q_{cc} =$

Q_{cl} and Q_{hl} = Q_{hc} = Q_{hs}, where Q_c is the heat load at the device cold side. Therefore, the I-dependent cooling temperature difference ($\Delta T = T_h - T_c$) at $Q_c = 0$ and the I-dependent cooling power density ($q_c = Q_c/A_s$) at $\Delta T = 0$ can be solved.

First, the influence of H on the cooling performance was studied with f fixed at 64%. Supplementary Figure 8a-d plots the I-dependence of ΔT and q_c at different H, where all electrical and thermal contact resistances are neglected or the reasonable r_{ec} of 1.0×10^{-10} $\Omega\cdot m^2$ and r_{tc} of 2×10^{-6} $m^2\cdot K\cdot W^{-1}$ are assumed. The H-dependent maximum cooling temperature difference (ΔT_{max}) and power density (q_{cmax}) can be extracted from Supplementary Fig. 8a-d, as shown in Supplementary Fig. 8e,f. It can be seen that when the contact resistances are ignored, q_{cmax} increases monotonically as H decreases, whereas ΔT_{max} rapidly decays when H is less than 50 μm due to the existence of electrode resistance and substrate thermal resistance. When considering the actual case when the contact resistances inevitably exist, both ΔT_{max} and q_{cmax} decrease overall. Moreover, q_{cmax} shows a non-monotonic H-dependence with a peak appearing at $H \approx 20$ μm, and ΔT_{max} exhibits a rapid decay earlier when H decreases below 100 μm. Therefore, it is very important to choose an appropriate thickness (~ 100 μm) of TE thick films to achieve a comprehensive cooling performance with large ΔT_{max} and q_{cmax}. Next, H was assumed to be 100 μm, and the influence of f on the cooling performance was calculated with the contact resistances considered (Supplementary Fig. 9). ΔT_{max} is only slightly affected by f, whereas q_{cmax} increases significantly as f. This indicates that achieving the highest possible filling factor is an effective way to improve the cooling performance of TECs. It is noteworthy that ΔT_{max} shows a weak non-monotonic dependence on f. This interesting result can be understood by analyzing the effect of electrode resistance and substrate thermal resistance on the ΔT_{max}-f dependence (the inset in Supplementary Fig. 9). As the device filling factor increases, an increase in the parasitic thermal resistance (due to the reduction in substrate size)

suppresses ΔT_{max}, while a decrease in the parasitic electrical resistance (attributable to the reduction in electrode size) enhances ΔT_{max}. Consequently, the competition between the two effects leads to the non-monotonic dependence of ΔT_{max} on *f*. In this study, we proposed a novel phase-change batch transfer strategy to integrate thick film μ-TECs with high filling factor.

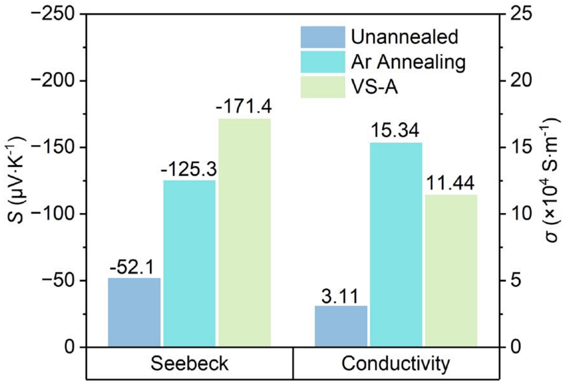

Supplementary Figure 1. Comparison of Bi_2Te_3 thermoelectric (TE) thick films obtained under different annealing conditions (unannealed, traditional Ar annealing in a tube furnace, and VS-A) on the room-temperature Seebeck coefficient (*S*) and electrical conductivity (*σ*).

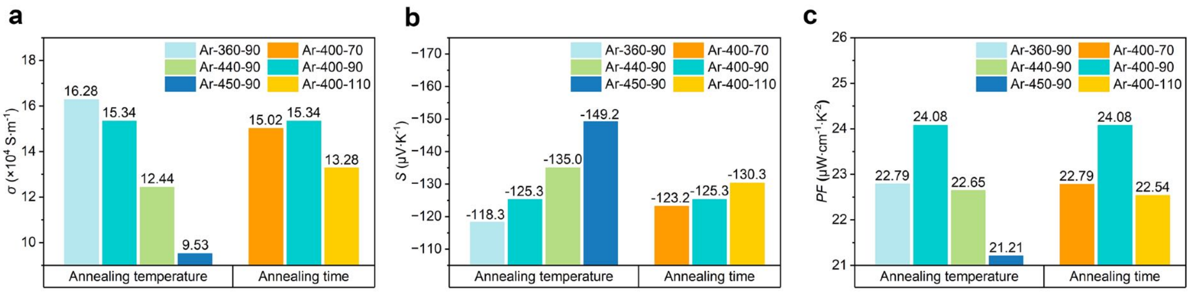

Supplementary Figure 2. TE properties of Bi_2Te_3 thick films prepared by traditional Ar annealing at different annealing temperatures (360, 400, 440, and 450 °C) and times (70, 90, and 110 min). a Electrical conductivity (*σ*). **b** Seebeck coefficient (*S*). **c** Power factor (*PF*).

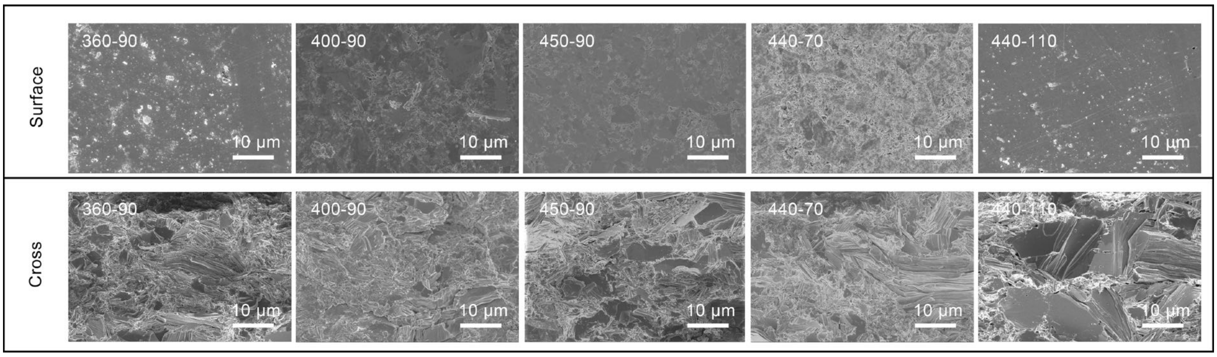

Supplementary Figure 3. SEM images of the surface and cross-sectional microstructure of Bi_2Te_3 thick films prepared by the powder direct molding method at different annealing temperatures (360, 400, 440, and 450 °C) and times (70, 90, and 110 min).

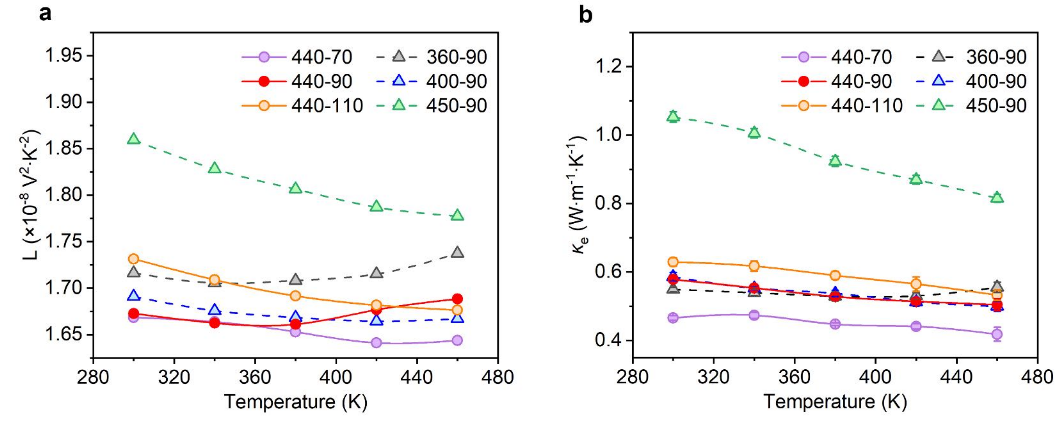

Supplementary Figure 4. Temperature-dependent electronic thermal properties of Bi_2Te_3 thick films prepared under different annealing conditions. a Lorenz Coefficient (*L*). **b** Electronic thermal conductivity (κ_e). The *L* and κ_e are calculated from the measured electrical conductivity and Seebeck coefficient. Data shown are mean +/- S.E.M. of three independent measurements (n = 3).

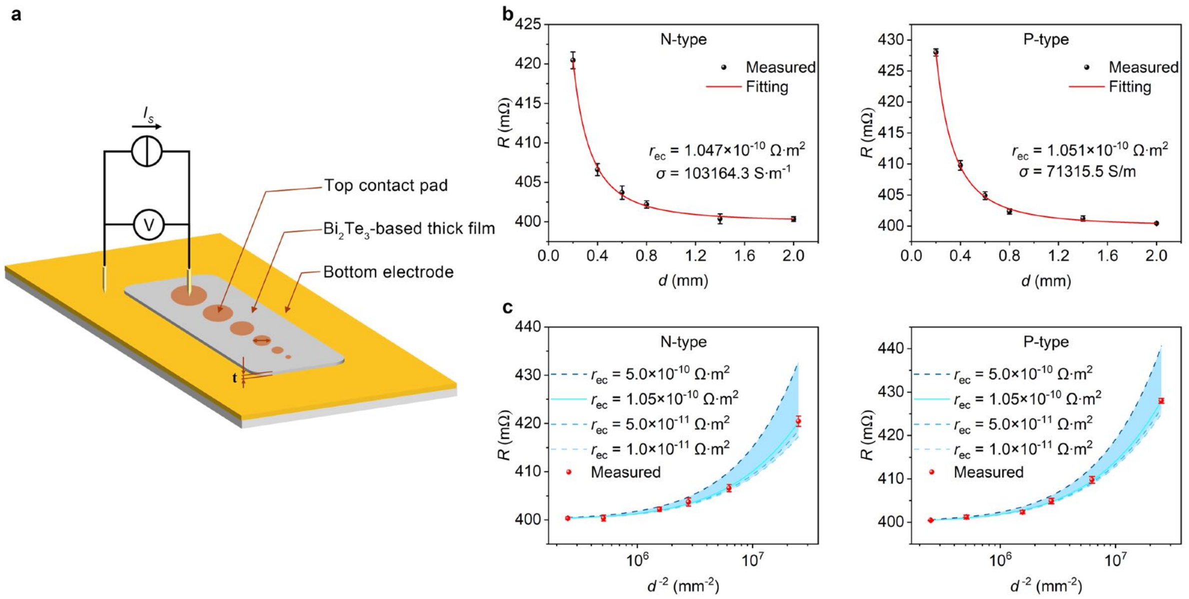

Supplementary Figure 5. Measurements of the out-of-plane electrical conductivity (σ) of Bi_2Te_3-based TE thick films and the electrical contact resistivity (r_{ec}) between the thick films and electrodes using the Cox-Strack method. **a** Schematic illustration of the test structure in the Cox-Strack method. **b** Measured resistance (black dots) between the bottom electrode and top contact pad as a function of pad diameter (d). The σ and r_{ec} for the N-type and P-type TE thick films are extracted from the fitting (red curve) of the Cox-Strack equation. **c** Resistance as a function of the inverse square pad diameter (d^{-2}). The light blue background indicates the impact of r_{ec} on the resistance. Data shown are mean +/- S.E.M. of three independent measurements (n = 3).

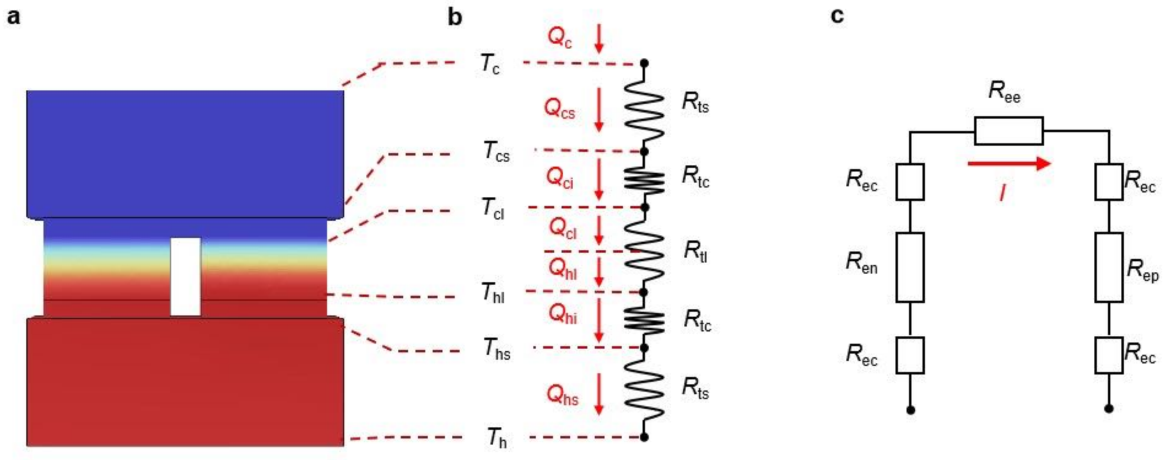

Supplementary Figure 6. Simplified one-dimensional model of the thermoelectric cooler (TEC). a Schematic illustration of a TEC containing a pair of TE legs. **b** One-dimensional equivalent thermal circuit of the TEC. **c** One-dimensional equivalent electrical circuit of the TEC.

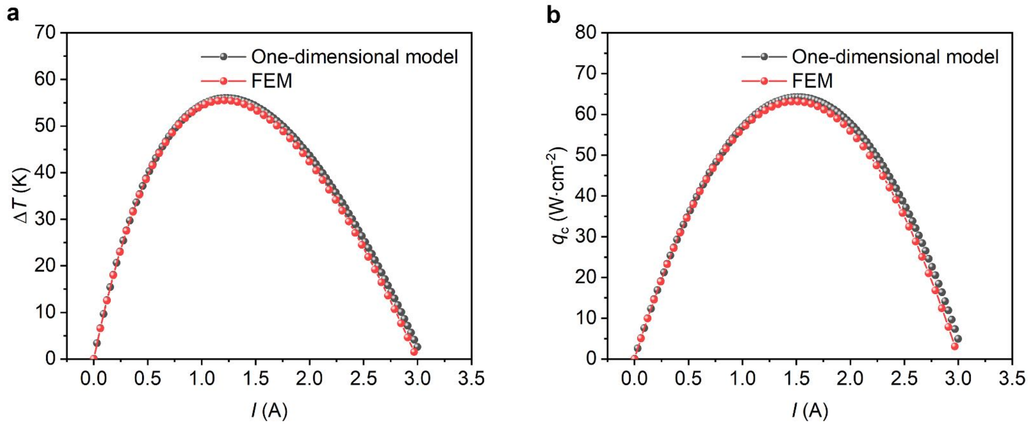

Supplementary Figure 7. Comparison between the one-dimensional model and finite element method (FEM) simulations under the conditions with a hot-side temperature of 25 °C, a leg height of 100 μm, and a device filling factor of 64%. a. Cooling temperature difference (ΔT) as a function of current. **b.** Cooling power density (q_c) as a function of current.

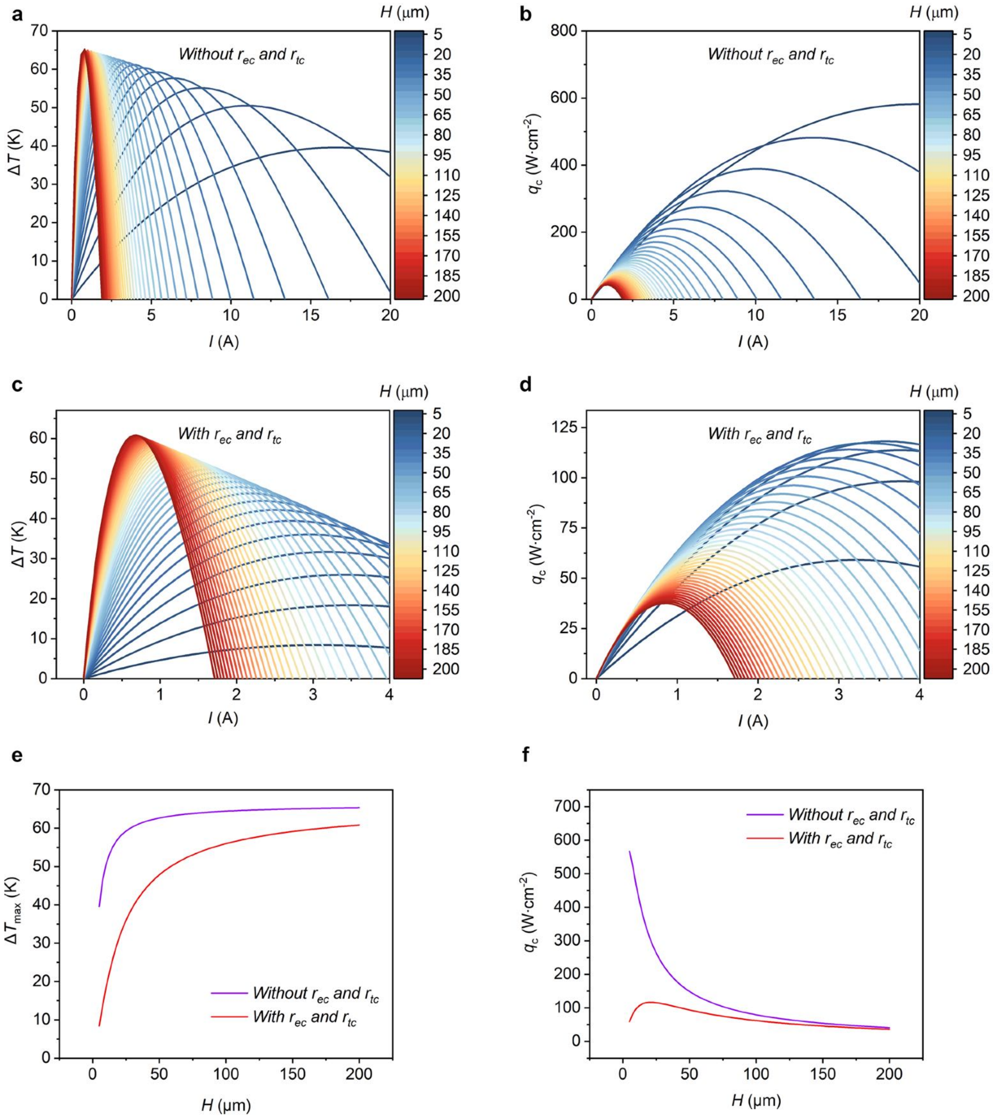

Supplementary Figure 8. Influence of TE leg height (*H*) on the cooling performance of the TEC under the conditions with a hot-side temperature of 25 °C and a device filling factor of 64%. a, b Cooling temperature difference (ΔT) and power density (q_c) as functions of current (I) at different H, while neglecting all electrical and thermal contact resistances. **c, d** ΔT and q_c as functions of I at different H, assuming a reasonable electrical contact resistivity ($r_{ec} = 1.0 \times 10^{-10}\ \Omega\cdot m^2$) and thermal contact resistivity ($r_{tc} = 2 \times 10^{-6}\ m^2\cdot K\cdot W^{-1}$) at the interface between the TE legs and electrodes. **e, f** Maximum cooling temperature difference (ΔT_{max}) and power density (q_{cmax}) extracted from a-d as functions of H. The conditions with or without contact resistances are compared.

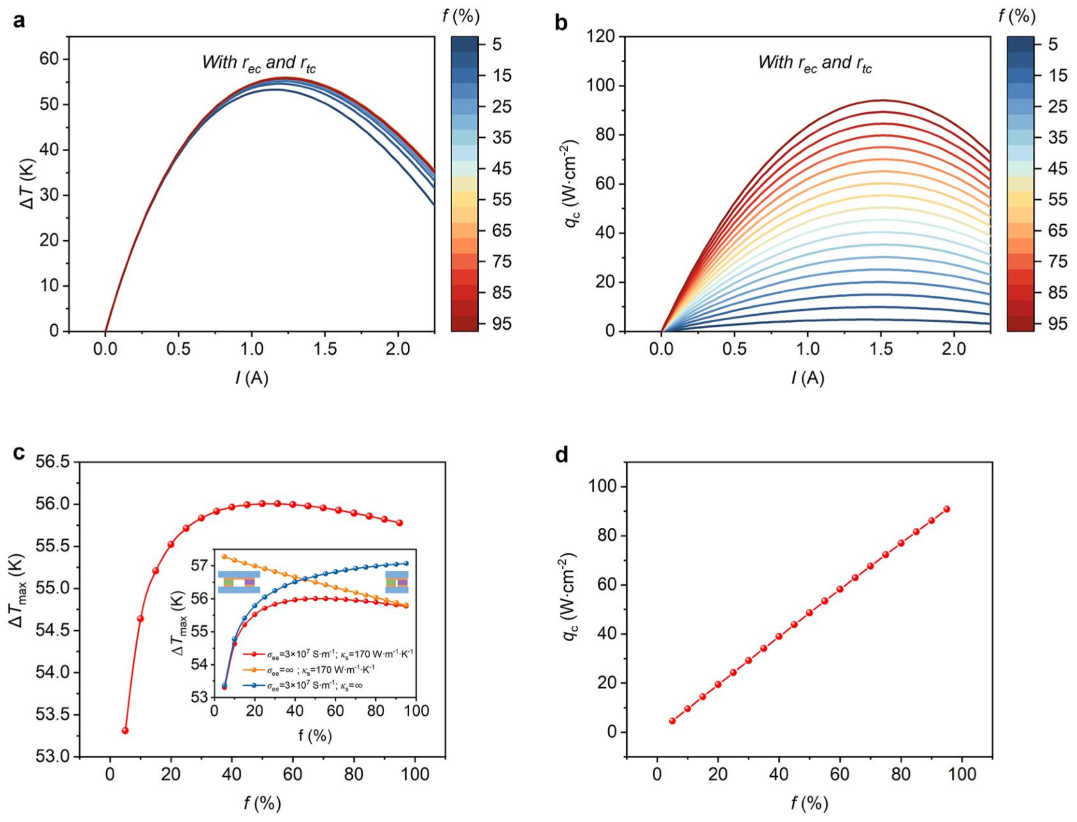

Supplementary Figure 9. Influence of device filling factor (f) on the cooling performance of the TEC under the conditions with a hot-side temperature of 25 °C and a leg height of 100 μm. a, b Cooling temperature difference (ΔT) and power density (q_c) as functions of current (I) at different f, assuming a reasonable electrical contact resistivity ($r_{ec} = 1.0 \times 10^{-10}$ Ω·m^2) and thermal contact resistivity ($r_{tc} = 2 \times 10^{-6}$ m^2·K·W^{-1}) at the interface between the TE legs and electrodes. **c** Extracted maximum cooling temperature difference (ΔT_{max}) as a function of f. The inset shows the effect of electrode resistance and substrate thermal resistance on the ΔT_{max}-f dependence. The red dot line takes into account the realistic electrode conductivity (σ_{ee}) and substrate thermal condutivity (κ_s). The orange dot line neglects the electrode resistance (σ_{ee}=∞). The blue dot line neglects the substrate thermal resistance (κ_s =∞). **d** Extracted maximum cooling power density (q_{cmax}) as a function of f.

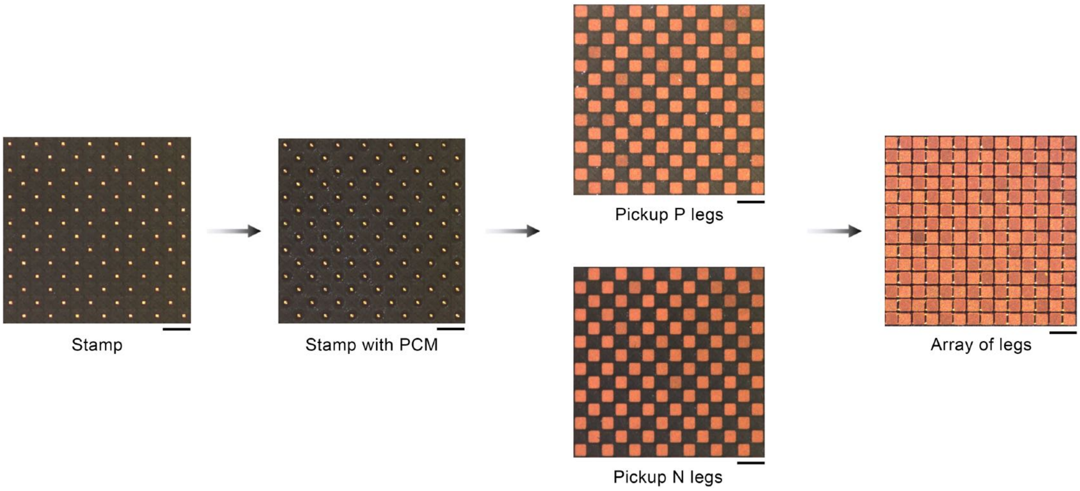

Supplementary Figure 10. Optical images illustrating the high-density phase-change batch transfer process of micro-components (TE legs with a size of 200 × 200 μm^2). First, 50 μm thick Cu on an aluminum nitride (AlN) substrate is etched to form a stamp with Cu convex points (a size of 50 × 50 μm^2). After coating the phase change material on convex points, the stamp is used to pickup P-type or N-type TE legs. Finally, by aligning and then alternately releasing the P-type and N-type TE legs on a receiving substrate, a high-density leg array can be formed. The rosin is chosen as the phase change material. The scale bar in the figure represents a length of 500 μm.

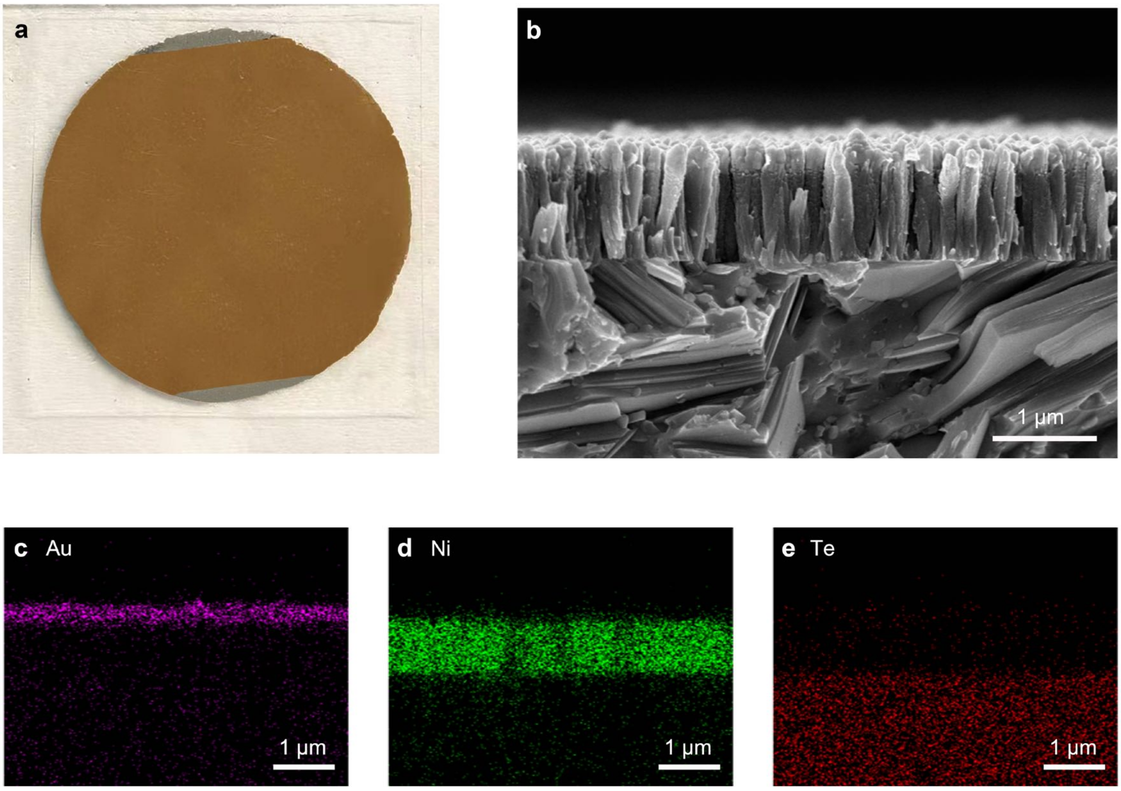

Supplementary Figure 11. Surface-metallized TE thick film. a Digital photo of a Bi_2Te_3 thick film with 1μmNi/200nmAu bilayer deposited on both sides. **b** SEM image of the sample cross-section. **c-e** Spatial distribution of different elements (Au, Ni, and Te) in the SEM image of b. There is no obvious element diffusion between the Au, Ni, and TE layers.

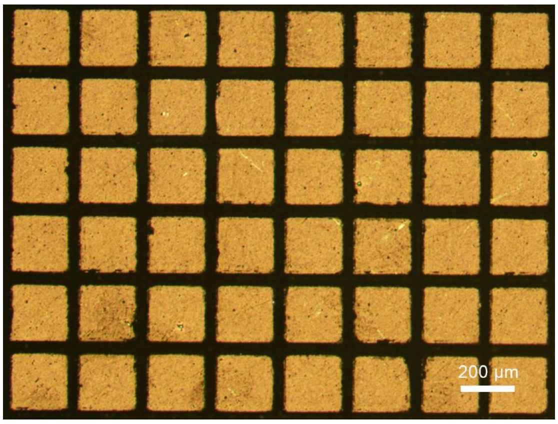

Supplementary Figure 12. Femtosecond laser cutting of the surface-metallized TE thick films. The thick films are cut into TE legs with a dimension of 200 × 200 µm^2, and the gap between adjacent TE legs is less than 50 µm due to the high laser processing precision.

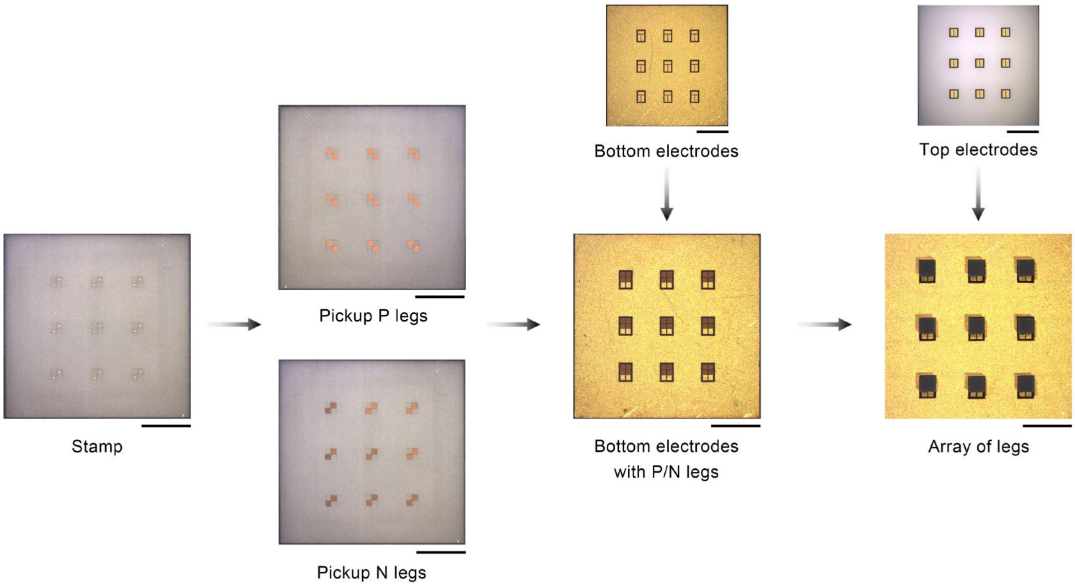

Supplementary Figure 13. Optical images illustrating the integration process of multiple μ-TECs on a single substrate at once. First, according to the electrode positions where TE legs need to be welded, a corresponding stamp with Cu convex points (a size of 50 × 50 μm^2) is prepared on an AlN substrate. After coating rosin on the convex points, P-type and N-type TE legs are picked up and alternately transferred to the PDMS substrate, followed by soldering with upper and lower electrodes. The scale bar in the figure represents a length of 2 mm.

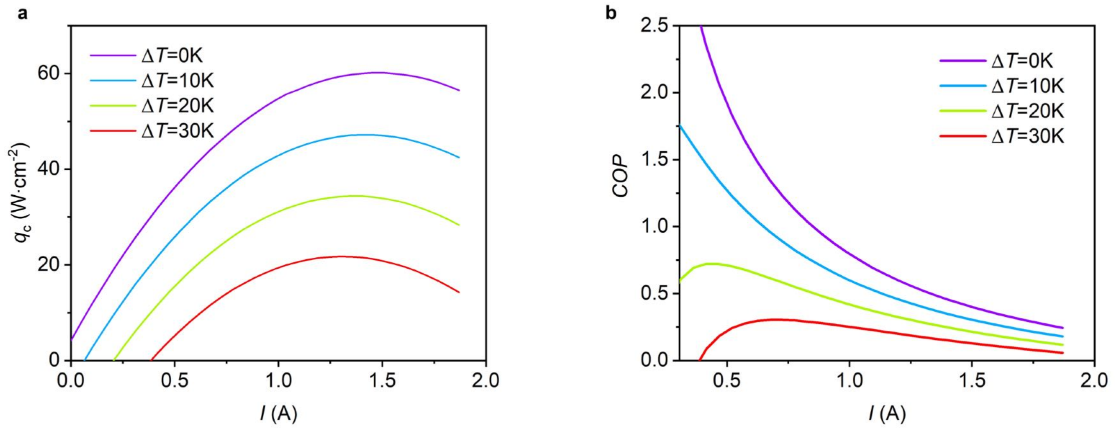

Supplementary Figure 14. FEM-simulated cooling performance of as-integrated thick-film μ-TECs with a size of 2.5 × 2.5 mm^2 as a function of current at various cooling temperature differences across the device. a Cooling power density (q_c). **b** Performance coefficient (*COP*).

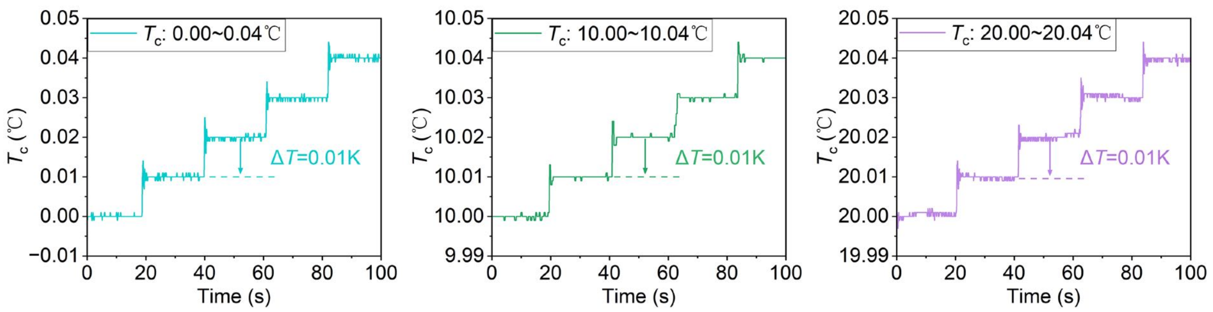

Supplementary Figure 15. Precise control of cold-side temperature (T_c) of the as-integrated thick-film μ-TECs over a wide temperature range from 0 °C to 20 °C. When the temperature is adjusted every 20 seconds in a step of 0.01 K, clear discernible temperature changes can be observed.

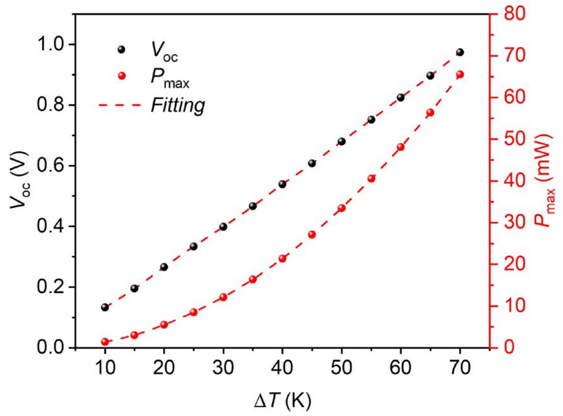

Supplementary Figure 16. Open-circuit voltage (V_{oc}) and maximum output power (P_{max}) as functions of the temperature difference (ΔT) applied between the cold side and hot side of the as-integrated thick-film μ-TEC. The V_{oc} and P_{max} exhibit linear and parabolic dependencies, respectively, with respect to the temperature difference, which is consistent with the theoretical predictions. The dashed lines are the linear and parabolic fittings.

Supplementary Table 1. Atomic percentage of Te and Bi contents in Bi_2Te_3 thick films prepared at different annealing temperatures (360, 400, 440, and 450 °C) and times (70, 90, and 110 min), characterized by the energy-dispersive X-ray spectroscopy.

Sample	Atomic percentage (%)		Te: Bi ratios
	Te	Bi	
360-90	59.84	40.16	2.98:2
400-90	59.27	40.73	2.91:2
440-70	59.01	40.99	2.88:2
440-90	58.76	41.24	2.85:2
440-110	58.25	41.75	2.79:2
450-90	57.71	42.29	2.73:2

Supplementary Table 2. Material (TE legs, electrodes, and subtrates) parameters used for the theoretical simulation of the cooling performance of TECs.

Material	σ ($S \cdot m^{-1}$)	κ ($W \cdot m^{-1} \cdot K^{-1}$)	S ($\mu V \cdot K^{-1}$)	PF ($\mu W \cdot cm^{-1} \cdot K^{-2}$)	ZT	Size (μm^2)	Thickness (μm)
N-type legs	103164.3	1.232	-173.40	31.02	0.75	200×200	-
P-type legs	71315.5	1.310	212.15	32.10	0.73	200×200	-
Electrodes	3×10^7	400	-	-	-	-	30
Substrates	-	170	-	-	-	-	200

Supplementary Table 3. Comparison of the as-integrated thick-film μ-TECs and reported polycrystalline Bi_2Te_3-based TE devices on maximum normalized voltage density and normalized power density, where the μ-TECs serve as a power generator[2-21].

	TE-leg height (μm)	Device area (mm^2)	ΔT (K)	V_{oc} (mV)	P_D (μW)	Normalized voltage density ($mV \cdot cm^{-2} \cdot K^{-1}$)	Normalized power density ($\mu W \cdot cm^{-2} \cdot K^{-2}$)
This work	100	6.25	70	974	65530	222.6	214.0
2	2000	231.88	380	290	60000	0.329	0.179
3	1600	1600	65	990	20500	0.95	0.303
4	3000	420	170	290	189000	0.395	1.39
5	1000	1330	105	5000	2101	3.58	14.3
6	1000	100	150	2400	185	16	8.22
7	500	300	50	90	11.4	0.6	1.52
8	650	1600	25.6	693	101	1.69	9.64
9	1000	50	14	8	1.2	1.14	11.7
10	135	96.04	38.6	220	2338	3	1.6338
11	200	84.64	21	34	3.3856	1	0.009
12	650	1600	25	500	78100	1	7.648
13	3	400	62.8	2600	1200	10.35	0.076
14	0.007	25	16	3.2	0.0013	94.6	0.0046
15	9	32.5	29.9	634	4660	65.2	16
16	10	28.6	39.7	320	1100	14	2.44
17	10	32.5	52.5	410	2990	12	3.338
18	100	300	1.5	3.69	0.2025	0.82	30
19	70	0.68	5	0.0065	0.6	17.6	38
20	20	25	22.3	294	5.9	0.026	0.04745
21	35	25	10	2300	2800	460	112

References

1. Cox, R. H. & Strack, H. Ohmic contacts for GaAs devices. *Solid-State Electron.* **10**, 1213-1218 (1967).
2. Chung, S.-H., Kim, J. T., Kim, H., Kim, J. & Kim, D. H. High-temperature Bi_2Te_3 thermoelectric generator fabricated using Cu nanoparticle paste bonding. *J. Alloy. Compd.* **896**, 163060 (2022).
3. Xu, Y. et al. Flexible and stretchable thermoelectric devices with Ni-EGaIn liquid metal electrodes for cooling and low-grade-body heat harvesting. *J. Alloy. Compd.* **945**, 169260 (2023).
4. Nguyen, Y. N. et al. Bulk Bi_2Te_3-based bendable thermoelectric device with highly elastic Cu-Be alloy foils. *Mater. Today Commun.* **34**, 105408 (2023).
5. Sugahara, T. et al. Fabrication with Semiconductor Packaging Technologies and Characterization of a Large-Scale Flexible Thermoelectric Module. *Adv. Mater. Technol.* **4**, 1800556 (2019).
6. Ekubaru, Y. et al. Fabrication and Characterization of Ultra-Lightweight, Compact, and Flexible Thermoelectric Device Based on Highly Refined Chip Mounting. *Adv. Mater. Technol.* **5**, 1901128 (2020).
7. Kim, S. J., We, J. H. & Cho, B. J. A wearable thermoelectric generator fabricated on a glass fabric. *Environ. Sci. Energy* **7**, 1959-1965 (2014).
8. Kim, S. J. et al. Post ionized defect engineering of the screen-printed $Bi_2Te_{2.7}Se_{0.3}$ thick film for high performance flexible thermoelectric generator. *Nano Energy* **31**, 258-263 (2017).
9. Park, S. H. et al. High-performance shape-engineerable thermoelectric painting. *Nat. Commun.* **7**, 13403 (2016).

10. Roth, R. et al. Design and Characterization of Micro Thermoelectric Cross-Plane Generators With Electroplated Bi_2Te_3, Sb_xTe_y, and Reflow Soldering. *J. Microelectromech. Syst.* **23**, 961-971 (2014).
11. Trung, N. H., Van Toan, N. & Ono, T. Fabrication of π-type flexible thermoelectric generators using an electrochemical deposition method for thermal energy harvesting applications at room temperature. *J. Micromech. Microeng.* **27**, 125006 (2017).
12. Andzane, J. et al. Thickness-dependent properties of ultrathin bismuth and antimony chalcogenide films formed by physical vapor deposition and their application in thermoelectric generators. *Mater. Today Energy* **19**, 100587 (2021).
13. Kim, S. J. et al. High-Performance Flexible Thermoelectric Power Generator Using Laser Multiscanning Lift-Off Process. *ACS Nano* **10**, 10851-10857 (2016).
14. Liu, Z. et al. High-performance integrated chip-level thermoelectric device for power generation and microflow detection. *Nano Energy* **114**, 108611 (2023).
15. Zhang, J. et al. Flexible micro thermoelectric generators with high power density and light weight. *Nano Energy* **105**, 108023 (2023).
16. Shin, K.-J. & Oh, T.-S. Micro-Power Generation Characteristics of Thermoelectric Thin Film Devices Processed by Electrodeposition and Flip-Chip Bonding. *J. Electron. Mater.* **44**, 2026-2033 (2015).
17. Zhang, W., Yang, J. & Xu, D. A High Power Density Micro-Thermoelectric Generator Fabricated by an Integrated Bottom-Up Approach. *J. Microelectromech. Syst.* **25**, 744-749 (2016).
18. Yang, Q. et al. Flexible thermoelectrics based on ductile semiconductors. *Science* **377**, 854-858 (2022).

19. El Oualid, S. et al. Innovative design of bismuth-telluride-based thermoelectric micro-generators with high output power. *Energy Environ. Sci.* **13**, 3579-3591 (2020).
20. Kim, M.-Y. & Oh, T.-S. Thermoelectric Thin Film Device of Cross-Plane Configuration Processed by Electrodeposition and Flip-Chip Bonding. *Mater. Trans.* **53**, 2160-2165 (2012).
21. Bottner, H., Nurnus, J., Schubert, A. & Volkert, F. New high density micro structured thermogenerators for stand alone sensor systems. In *Proc. 26th International Conference on Thermoelectrics* 306-309 (IEEE, 2007).
